# Probing the composition of *Plasmodium species* contained in malaria infections in the Eastern region of Ghana

**DOI:** 10.1186/s12889-019-7989-1

**Published:** 2019-12-02

**Authors:** Linda Eva Amoah, Dickson Donu, Benjamin Abuaku, Colins Ahorlu, Daniel Arhinful, Edwin Afari, Keziah Malm, Kwadwo Ansah Koram

**Affiliations:** 1 Immunology Department, Noguchi Memorial Institute for Medical Research, University of Ghana, Accra, Ghana; 2 West Africa Center for Cell biology of Infectious Pathogens (WACCBIP), College of Basic and Applied Sciences, University of Ghana, Accra, Ghana; 3 Epidemiology Department, Noguchi Memorial Institute for Medical Research, University of Ghana, Accra, Ghana; 4 School of Public Health, University of Ghana, Accra, Ghana; 5 National Malaria Control Program, Accra, Ghana

**Keywords:** Plasmodium, Malariae, Ovale, Falciparum, RDT, Submicroscopic

## Abstract

**Background:**

Asymptomatic *falciparum* and non-*falciparum* malaria infections are major challenges to malaria control interventions, as they remain a source of continual infection in the community. This becomes even more important as the debate moves towards elimination and eradication. This study sought to quantify the burden of *Plasmodium* malaria infection in seven communities in the Eastern Region of Ghana.

**Methods:**

The cross-sectional study recruited 729 participants aged 85 years old and below from 7 closely linked communities. Finger pricked blood was used to prepare thick and thin blood smears as well as spot filter paper and an histidine rich protein 2 (HRP2) rapid diagnostic test kit (RDT). Genomic DNA was extracted from the filter paper dry blood spot (DBS) and used in PCR to amplify the *Plasmodium* 18S rRNA gene using species specific PCR.

**Results:**

96.6% of the participants were identified as afebrile, with axillary temperatures below 37.5 °C. PCR identified 66% of the participants to harbor malaria parasites, with 9 *P. malariae* and 7 *P. ovale* mono-infections accounting for 2.2% and *P. falciparum* combined with either 36 *P. malariae* or 25 *P. ovale* infections, accounting for 13.3%. Parasite prevalence by microscopy (32%) was similar to the RDT positivity rate (33%). False positive RDT results ranged from 64.6% in children aged between 5 and 9 years to 10% in adults aged 20 years and above. No significant differences were observed in *falciparum* and non-*falciparum* parasite carriage at the community level, however young adults aged between 15 and 19 years had the highest prevalence (34.8% (16/46)) of *P. falciparum* and *P. malariae* parasite carriage whilst children aged between 5 and 9 years had the highest level (11.4% (14/123)) of *P. ovale* carriage.

**Conclusion:**

The high rate of misidentification of non-*falciparum* parasites and the total absence of detection of *P. ovale* by microscopy suggests that more sensitive malaria diagnostic tools including molecular assays are required to accurately determine the prevalence of carriers of non-*falciparum* parasites and low density *P. falciparum* infections, especially during national surveillance exercises. Additionally, malaria control interventions targeting the non-*falciparum* species *P. malariae* and *P. ovale* parasites are needed.

## Background

Malaria, a parasitic disease caused in humans by five different species of the *Plasmodium* genus namely *P. falciparum*, *P. malariae*, *P. ovale*, *P. vivax* and *P. knowlesi*, remains a devastating public health problem in the tropical and subtropical regions worldwide [1]. The burden of malaria in Ghana remains high, despite the decrease in malaria related deaths from 2985 to 1264 in 2016 [2]. Notwithstanding the slowdown in the prevalence of symptomatic malaria [1], afebrile carriage of malaria parasites is a huge problem in a number of malaria endemic countries, mainly because the parasite carriers do not exhibit any of the clinical symptoms of malaria and are thus untreated [3]. Since gametocytes, the transmissible forms of the malaria parasite are produced at each erythrocytic cycle of the parasite these asymptomatic carriers continuously serve as transmission reservoirs [4] until they are cured of their infection.

In sub-Saharan African, the majority of malaria cases are caused by *P. falciparum* [1] with a minor but underestimated prevalence of other *Plasmodium* species [5]. In Ghana, *P. falciparum* is the most prevalent malaria causing species with a prevalence of 98% followed by *P. malariae* and *P. ovale* with prevalence of 2–9 and 1% respectively [6]. A study by Owusu et al in 2017, reported *P. malariae* prevalence of 12.7% in the Kwahu south Region of Ghana [5] as compared to the national *P. malariae* prevalence of 2–9% [6]. The global distribution of *P. malariae* is sparse and variable, but is similarly endemic to West Africa, and other malaria endemic areas of the world [7–9]. *Plasmodium malariae* infections usually present as asymptomatic infections although some may result in clinical disease state [10, 11]. The distribution of *P. ovale* is relatively limited but highly prevalent in tropical areas of Africa, including sub-Saharan Africa [12]. *Plasmodium vivax* is endemic in Asia but scarce in West Africa where the natives lack the Duffy antigen receptor for chemokines, an essential receptor for erythrocyte invasion by *P. vivax* [13]. Despite the widespread absence of the Duffy antigen receptor for chemokines in people from sub Saharan Africa a few recent reports of have identified *P. vivax* infections in some sub Saharan African countries including Mali and Nigeria [14–16].

In order to implement accurate measures for effective control and treatment of malaria the detection of all human *Plasmodium* species is important [17]. Recently, the definition of malaria elimination has been revised to include the interruption of the local transmission of all human malaria parasites [18], making it necessary that national control programs include surveillance of all malaria parasite species. However, due to the very low occurrence and parasite densities of *P. malariae* and *P. ovale* in sub-Saharan Africa where *P. falciparum* is endemic, only a few microscopists are able to correctly identify *P. malariae* and *P. ovale* infections [19]. This combined with the fact that most *P. malariae* and *P. ovale* infections present as mixed infections with *P. falciparum* [11, 20], likely contribute to the misdiagnosis and the very low reported prevalence of other *Plasmodium* species in populations where *P. falciparum* is highly endemic. Rapid diagnostic test kits for malaria have improved malaria diagnostics, however this is mainly true for *P. falciparum* infections where the histidine rich protein 2 (HRP2) antigen, which is specific for *P. falciparum,* is detected as these kits have the highest sensitivities compared with the other rapid diagnostic kits for malaria [21]. Detection of parasite lactate dehydrogenase and aldolase antigens can be used for the detection of all *Plasmodium* species either combined as a pan specific test kit (aldolase) or separately using species-specific lactate dehydrogenase, and these have higher specificities than the HRP2 based tests but are less sensitive [21].

In this study we sought to determine whether the contribution of *P. malariae* and *P. ovale* to the overall prevalence of malaria captured during multiple community surveys in the Eastern Region of Ghana could be accurately predicted without the use of molecular tools, especially as numerous community surveys are carried out without the use of molecular tools. As such we used microscopy, RDT and species-specific PCR to assess the prevalence and composition of malaria parasites carried by consenting individuals living in seven closely linked communities in the Eastern Region of Ghana.

## Methods

### Characteristics of study site

Pakro is one of the five sub-districts in the Akwapim South Municipal district of the Eastern Region of Ghana. Pakro comprises of 22 communities and has an estimated population of 7655 with and is located in the Akwapim South district in the Eastern region of Ghana [22]. There are two major rainy seasons, May to June and September to October. Parasite positivity rate by RDT in 2014 was 47.5% [23] and was identified by microscopy in 2017 to be 27.5% (unpublished data). The Pakro Health Center, which is the major Health Center serving the sub district and its surrounding communities is one of the 30 National Malaria Control Program sentinel sites monitoring malaria prevalence in Ghana. In 2017 adherence by the Pakro Health Center to the test treat and tract policy was 100% [24].

### Sample size and sampling method

A minimum sample size of 675 individuals of all ages was computed for the study based on an estimated overall malaria prevalence of not less than 25% at 95% confidence interval, precision of 4% and design effect of 1.5. A total of 15 compounds were randomly selected in each of the 7 communities to be able to achieve the minimum sample size based on an estimated average compound size of 6.4 in the Akwapim south district as per the 2010 population and housing census [22]. All residents in the selected compounds were eligible for testing.

### Study design

**Fig. 1:**
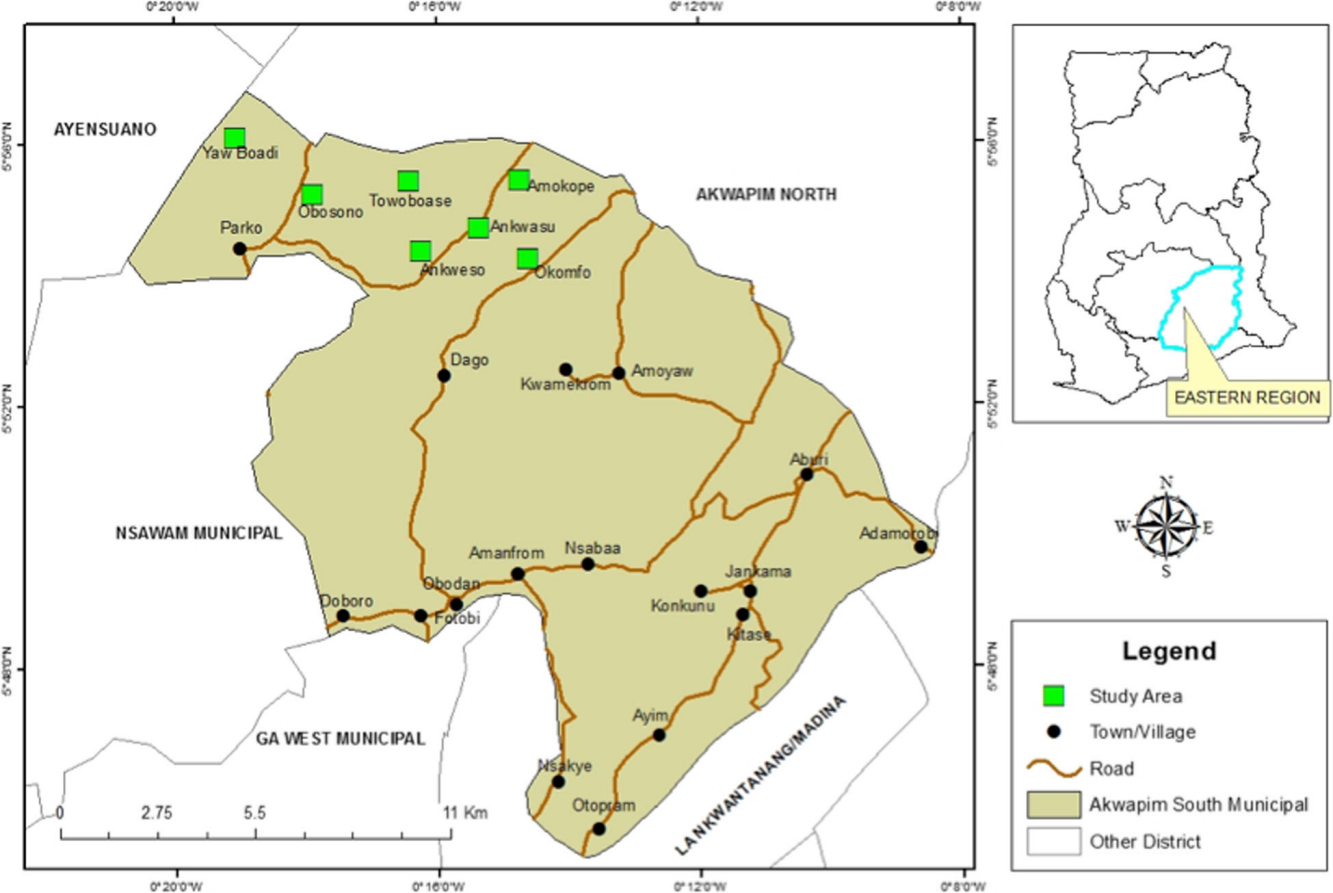
A map of Ghana highlighting study sites located within the Eastern Region

This cross-sectional study was conducted in 7 (Amokope (AM), Ankwensu (AN), Anwensu (AW), Obosono (OB), Okomfo (OK), Towoboase (TO) and Yaw Boadi (YB)) out of 22 communities within the Pakro sub-district (Fig. 1), which were not included in an implementation research on malaria mass testing and treatment.

### Sample collection and processing

Blood samples were obtained from a total of 729 afebrile individuals of all ages between October and November 2017. A finger prick sample of whole blood (~ 100 μl) was obtained from each individual. The blood was used to spot a Whatman No. 3 filter paper as well as SD Bioline HRP2 rapid diagnostic kit following the manufacturer’s instruction. Thick and thin blood smears were also prepared for each individual. The blood stained Whatman No. 3 filter papers were air-dried and individually stored in a desiccated Ziploc bag. The thin smears were fixed in methanol and stained, together with the thick smears, in 3% Giemsa stain for 30–45 min. The stained slides were rinsed, air-dried, and stored in plastic slide boxes for reading. All the microscopists were blinded of the RDT results.

### Malaria parasite identification and quantification by microscopy

Parasite quantification was done using the thick smears whilst the thin smears were used for species identification. *Plasmodium* parasite density (PD) was estimated by multiplying the number of parasites identified per 200 white blood cells by 40, given that there are approximately 8000 white blood cells/μl of blood. A thick smear was considered negative for *Plasmodium* parasites if no parasites were observed in 200 high-powered fields. Each blood smear was read by 2 independent microscopists. Discordant smear readings in terms of the presence of asexual/sexual stage parasites as well as species identification were re-examined by a third microscopist. Discordant results agreeing with the third reading were considered final.

### DNA extraction

Parasite DNA was extracted from the dried filter paper blood spots using the Chelex extraction method as previously described [25, 26]. Each filter paper dried blood spot (DBS) was punched into a sterile 1.5 ml microfuge tube containing 1 ml 1X phosphate buffered saline (PBS) at a pH of 7.4 supplemented with 50 μl of a 10% saponin solution. The tubes were incubated at 4 °C overnight. The DBS punches were washed for 30 min in ice cold PBS at 4 °C and the supernatant discarded. Finally, 60 μl of freshly prepared 20% Chelex-100 in 1X PBS and 140 μl of distilled water was added to each tube. The tubes were then heated at 95 °C for 10 min to extract the DNA from the samples. The tubes were finally centrifuged at 13000 rpm for 6 min and 120 μl of the supernatant transferred into a new sterile labeled 0.5 ml microfuge tube. The DNA was stored at − 20 °C or used immediately.

### *Plasmodium species (P. falciparum*, *P. malariae* and *P. ovale*) identification

Nested PCR was used to amplify unique regions of the *P. falciparum*, *P. malariae* and *P. ovale* 18 s rRNA gene from genomic DNA extracted from the samples using a previously described protocol [27] with little modifications. In the primary reaction, 80 nM of the genus specific primers rPLU5 (forward) and rPLU6 (reverse) were used in a total reaction volume of 15 μl made up of 5 μl DNA template, 1X PCR buffer, 167 nM dNTPs, 2.5 mM MgCl_2_ and 1 U of OneTaq DNA polymerase. In the secondary reaction (nest 2), the species-specific primers rFAL1/rFAL2 (133.33 nM), rMAL1/rMAL2 (333.33 nM) and rOVA1/rOVA2 (333.33 nM) were used in separate 15 μl reactions for the identification of *P. falciparum*, *P. malariae* and *P. ovale* respectively. The template for the secondary reaction was 0.5 μl of the primary reaction product. Details of all the primers used are provided in Additional file 1: Table S1.

The primary and nested PCR reaction cycling conditions included an initial denaturation at 95 °C for 5 min followed by 35 cycles a second denaturation at 94 °C for 30 s, primer annealing step at 55 °C (for primary) or 58 °C (for nested) for 1 min and sequence extension at 68 °C for 1 min and a final extension at 68 °C for 5 min.

The secondary PCR products were resolved on a 1% agarose gel pre-stained with ethidium bromide, which were subsequently visualized using the UV settings on a Vilbar gel documentation system.

### Data analysis

Data were either considered as a complete dataset or grouped according to collection community or categorized into young children below 5 years, children between 5 and 9 years, older children between 10 to 14 years, young adults 15 to 19 years and adults (20 years and above). Prevalence of *Plasmodium* infections was calculated as the proportion of individuals that were identified as positive for the presence of parasites (either all parasite species or an individual species) in each community or age group category.

The Kruskal-Wallis test (GraphPad Prism ver 5) was used to determine significant differences between the median ages of the study participants in each community. GraphPad Prism ver 5 was also used to determine the mean PD and its standard error (SEM) in the different sites and cohorts. Descriptive statistics including frequency and Pearson Chi-Square analysis was used to identify significant differences between parasite prevalence in the different sites and cohorts (IBM SPSS ver 22). *P* values of less than 0.05 were considered statistically significant.

## Results

The study population comprised of 729 participants aged below 85 years (Table 1) recruited from 7 closely linked communities. The proportion of males in the study population ranged between 37% (17/46) in young adults between 15 years to 19 years and 60% (74/123) in children between 5 years to 9 years (Table 1). A total of 3.4% (25/729) of the study participants were identified as febrile and had axillary temperatures of or above 37.5 °C. Children aged between 5 and 9 years had the highest prevalence of febrile cases (5.6%) and adults ≥20 years old had the least prevalence of febrile cases (2.4%) (Table 1). There was no significant difference (Kruskal-Wallis test, *p* = 0.6621) in the median ages of the participants from all the 7 communities.

**Table 1:** Demographic characteristics of study participants

	< 5 yrs. (79)	5–9 yrs. (123)	10–14 yrs. (112)	15–19 yrs. (46)	≥20 yrs. (369)	Total (729)
Sex (M/F)	40/39	74/49	58/54	17/29	160/207	349/378
Febrile	2 (2.5)	7 (5.6)	5 (4.5)	2 (4.4)	9 (2.4)	25 (3.4)

*M* male, *F* female, *yrs*. years, Febrile, axillary temperature ≥ 37.5 °C

### Parasite prevalence

#### Rapid diagnostic test (RDT)

About a third of the population sampled, 33.1% (241/729), tested positive for *P. falciparum* using the HRP-2 RDT kit. Parasite positivity rates were significantly different (Pearson Chi-Square = 145.557, *p* = 0.000) across the five age groups with the lowest prevalence identified in adults 20 years old and above (13.8% (51/369)) and highest prevalence (64.2% (79/123)) in children aged between 5 and 9 years old (Table 2). Parasite positivity rates were significantly higher in Obosono (Pearson Chi Square 6.494, *p* = 0.039) with a rate of 41.5% (50/122) and lowest positivity rate was 23.7% (18/76) in Amokope (Table 3).

**Table 2:** Age stratified prevalence of malaria in study participants

	< 5 yrs. 79	5–9 yrs. 123	10–14 yrs. 112	15–19 yrs. 46	≥20 yrs. 379	Total 729
RDT	38 (48.1)	79 (64.2)	60 (53.6)	13 (28.3)	51 (13.5)	241 (33)
Microscopy Positives (All Species)	30 (38)	67 (54.5)	58 (51.8)	13 (38.2)	65 (17.2)	233 (32)
Microscopy Mono	30 (100)	67 (100)	57 (98.3)	12 (92.3)	65 (100)	231 (99)
Microscopy Multi	0 (0)	0 (0)	1 (1.7)	1 (7.7)	0 (0)	2 (0.86)
Gametocytes	6 (7.6)	8 (6.5)	9 (8.04)	2 (4.3)	7 (1.9)	32 (4.4)
PD mean	9591.2	3837.4	3455.8	150.9	507.6	334.6
PD range (min - max)	47.7–113,462	16–68,000	15.1–95,555.6	16–553.4	3.8–9014.6	3.8–113,462
PCR Positive (All Species)	43 (54.4)	100 (81.3)	99 (88.4)	34 (73.9)	204 (53.8)	481 (66)
*Pf*	41 (95.4)	97 (97.0)	98 (99)	34 (100)	194 (51.2)	465 (96.7)
*Pm*	1 (2.3)	15 (15.0)	16 (16.2)	1 (2.9)	15 (7.4)	48 (10)
*Po*	5 (11.6)	14 (14.0)	5 (5.1)	1 (2.9)	10 (5.2)	35 (7.3)
PCR Mono	39 (90.7)	75 (75.0)	81 (81.8)	32 (92.1)	189 (92.7)	417 (86.7)
PCR Multi	4 (9.3)	25 (25.0)	18 (18.2)	2 (5.9)	15 (7.4)	64 (13.3)

*Mono* single parasite species infection, *multi* multiple parasite species infection, *RDT* PfHRP2 based malaria rapid diagnostic test, *PD* parasite density (parasites/μl) of blood, *Pf Plasmodium falciparum*, *Pm Plasmodium malariae*, *Po Plasmodium ovale*, *yrs.* years. Values are written as counts (%)

**Table 3:** Parasite prevalence by RDT, microscopy and PCR

	OB (122)	TB (132)	OK (94)	AK (117)	AW (134)	AM (76)	YB (54)	Total (729)
PCR Positive (%)	87 (71.3)	74 (56.1)	62 (66.0)	81 (69.2)	91 (67.9)	46 (60.5)	40 (74.1)	481 (66.0)
< 5 yrs. (%)	8/12 (66.7)	4/14 (28.6)	5/7 (71.4)	12/17 (70.6)	5/11 (45.5)	5/11 (45.5)	4/7 (57.1)	43/79 (54.4)
5–9 yrs. (%)	19/25 (76)	17/20 (85)	12/17 (70.6)	14/16 (87.5)	22/27 (81.5)	8/9 (88.9)	8/9 (88.9)	100/123 (81.3)
10–14 yrs. (%)	19/21 (90.5)	23/24 (95.8)	15/19 (78.9)	12/14 (85.7)	21/ 23 (91.3)	2/3 (66.7)	7/8 (87.5)	99/112 (88.4)
15–19 yrs. (%)	7/9 (77.8)	3/9 (33.3)	6/8 (75)	5/5 (100)	10/12 (83.3)	0/0	3/3 (100)	34/46 (73.9)
≥20 yrs. (%)	34/55 (61.8)	27/65 (41.5)	24/43 (55.8)	38/65 (58.5)	33/61 (54.1)	31/53 (58.3)	18/27(66.7)	205/369 (55.6)
Microscopy								
Positive (%)	42 (34.4)	48 (36.4)	38 (40.4)	37 (31.6)	38 (28.4)	19 (25.0)	11 (20.4)	233 (32.0)
Mono Pf	36 (29.5)	33 (25.0)	33 (35.1)	30 (25.6)	35 (26.1)	19 (25.0)	6 (11.1)	192 (26.3)
Mono Pm	6 (4.9)	15 (11.4)	6 (6.4)	8 (6.8)	3 (2.2)	0 (0)	5 (9.3)	43 (5.9)
Mono Po	0 (0)	0 (0)	0 (0)	0 (0)	0 (0)	0 (0)	0 (0)	0 (0)
Multispecies (%)	0 (0)	0 (0)	1 (1.1)	1 (0.9)	0 (0)	0 (0)	0 (0)	2 (0.3)
Febrile (≥37.5 °C)	2 (1.6)	4 (3.0)	2 (2.1)	10 (8.5)	2 (1.5)	5 (6.6)	0 (0)	25 (3.4)
PD (p/μl) Mean (SEM)	1685 (622.3)	2251 (1425)	3724 (2519)	3067 (979.7)	2575 (1043)	6322 (5955)	11,723 (7631)	3349 (823.6)
PD (p/μl) Min - max	14–17,359	16–68,000	15–95,556	16–23,360	15–31,849	4–113,462	16–68,000	4–113,462
RDT (%)	50 (41.0)	46 (34.8)	36 (38.3)	32 (27.6)	44 (32.8)	18 (23.7)	15 (28.9)	241 (33.1)

*OB* Obosono, *TB* Towoboase, *OK* Okomfo, *AK* Akwensu, *AW* Awensu, *AM* Amokope, *YB* Yaw Boadi, *RDT* PfHRP2 based malaria rapid diagnostic test positive samples, *PD* parasite density per μl blood, *min* minimum, *max* maximum, *Mono* single species infection, Febrile, axillary temperature (≥37.5 °C). PCR positive and microscopy positive samples include all *Plasmodium* species

#### Microscopy estimation of parasite prevalence and species

Approximately a third (233/729; 32.0%) of the participants were identified by microscopy to be positive for *Plasmodium* parasites. The agreement between first and second slide readings was approximately 96%. Almost 99% (231/233) of the infections identified by microscopy were single species infections, with *P. falciparum* accounting for 81.5% (190/231) and *P. malariae* accounting for 17.6% (41/232). Mixed species infections accounted for 0.85% (2/233) of the microscopic density infections. No sample was identified as containing *P. ovale* by microscopy.

Parasite prevalence was significantly different amongst the five different age groups (Pearson Chi-Square = 85.428, *p* = 0.000) and ranged from 17.6% in the adults aged 20 and above to 54.5% in children aged 5–9 years old. Gametocyte carriage amongst the different age groups was significantly different (Pearson Chi-Square = 12.255, *p* = 0.016). Gametocyte carriage was highest in the young adults (8% (9/112)), followed by the young children (7.6% (6/79)) and least in the adult (20 years and above) group (1.9% (7/369))(Table 2).

Parasite carriage was, similar across the seven sites (Pearson Chi-Square 10.450, *p* = 0.107). The highest 40.4% (38/94) and lowest 20.4% (11/54) prevalence of people with microscopic densities of parasites were recorded in Okomfo and Yaw Boadi respectively (Table 3). Similarly, gametocyte carriage amongst the sites was similar and ranged from 0% in Amokope to 7.4% (9/122) in Obosono.

#### Molecular (polymerase chain reaction, PCR) estimation of parasite prevalence and species

**Fig. 2:**
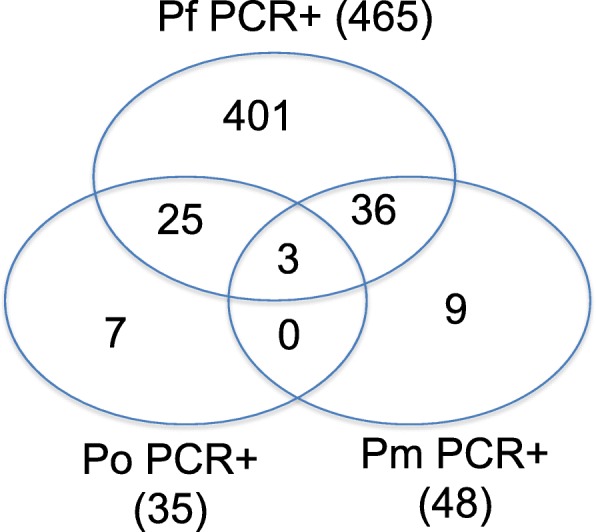
Prevalence of PCR detectable *Plasmodium* species within the study sites. A Venn diagram showing the frequency as counts of each of the *Plasmodium* species identified by PCR

Amplification of the *Plasmodium 18SrRNA* gene from the extracted DNA identified 66% (481/729) samples as positive for *Plasmodium* parasites (Tables 2 and 3). Out of the *Plasmodium* positive samples, 417 (86.6%) were mono-infections, with *P. falciparum* mono-infections accounting for 96.2% of the total (401/417) and *P. malariae* and *P. ovale* each contributing 2.1% (9/417) and 1.7% (7/417) to the mono-infections respectively. Combined, *P. falciparum* infections (mono and mixed) determined by PCR accounted for 96.67% (465/481) of the total *Plasmodium* positive samples. Multispecies-infections constituted 13.3% (64/481) of the infected samples, of which *P. falciparum* and *P. malariae* co-infections represented 56% (36/64) and *P. falciparum* and *P. ovale* represented 39% (25/64) of multi-infected samples. There were 5% (3/64) of the samples that tested positive for all three species, *P. falciparum, P. malariae* and *P. ovale* species. No co-infection of *P. malariae* and *P. ovale* in the absence of *P. falciparum* was identified (Fig. 2).

**Fig. 3:**
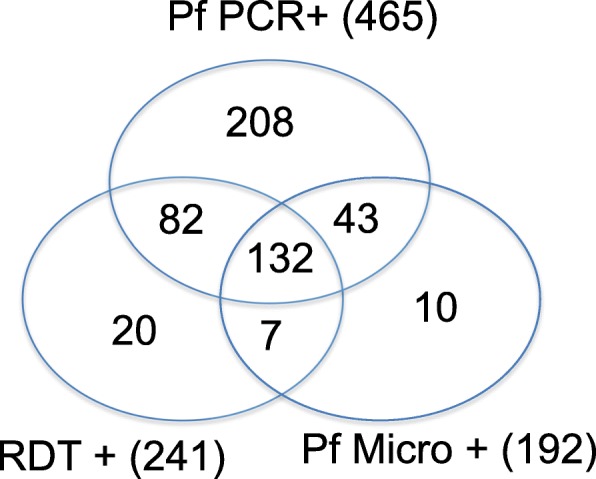
Prevalence of *P. falciparum* detected by varying diagnostic tools. A Venn diagram showing the detection of *P. falciparum* by microscopy, RDT and *P. falciparum* specific PCR

Out of the 465 samples that tested positive for *P. falciparum* by PCR, 251 (54%) and 290 (62.4%) tested negative (false negative) by RDT and microscopy (*P. falciparum*) respectively; 175 samples tested positive by both PCR and microscopy (*P. falciparum*), 214 samples tested positive by both PCR and RDT (Table 4) and 132 samples tested positive by all 3 *P. falciparum* specific tests (Fig. 3).

**Table 4:** Sensitivity and specificity of *Plasmodium falciparum* parasite detection

	Pf PCR+ (465)	Pf PCR- (264)
RDT+ (241)	214	27
RDT- (488)	251	237
Pf Micro+ (192)	175	17
Pf Micro- (537)	290	247

*Pf Plasmodium falciparum*, *micro* microscopy, *RDT* PfHRP2 based malaria rapid diagnostic test, +, positive; −, negative. The numbers in the table represent frequency in counts

Taking PCR as the reference, false positive results (samples that were negative by *P. falciparum* specific PCR) accounted for 11.2% (27/241) and 8.9% (17/192) of the RDT and *P. falciparum* positive microscopy samples respectively (Table 4).

False positive microscopy results were most (13.8% (8/58)) prevalent in older children aged between 10 and 14 years old and least (2.4% (1/41)) prevalent in young adults aged 15 to 19 years old. Whilst submicroscopic densities of *P. falciparum* estimated by PCR were most (81.7% (58/71)) prevalent in older children aged between 15 and 19 years old and least (35.2% (19/54)) prevalent in young children aged 4 years and below.

False positive RDT results were most (21.1% (8/38)) and least (0% (0/13)) prevalent in children aged 4 years old and below and young adults aged 15 to 19 years old respectively. Whilst false negative RDT results were most (78.8% (41/52)) and least (26.8% (11/41)) prevalent in older children aged between 10 and 14 years old and young children aged 4 years and below respectively.

Out of the 48 samples that tested positive for P*. malariae* by PCR, only 11 were identified by microscopy, whilst 32 samples that were identified as positive for *P. malariae* by microscopy were negative for *P. malariae* by PCR.

**Fig. 4:**
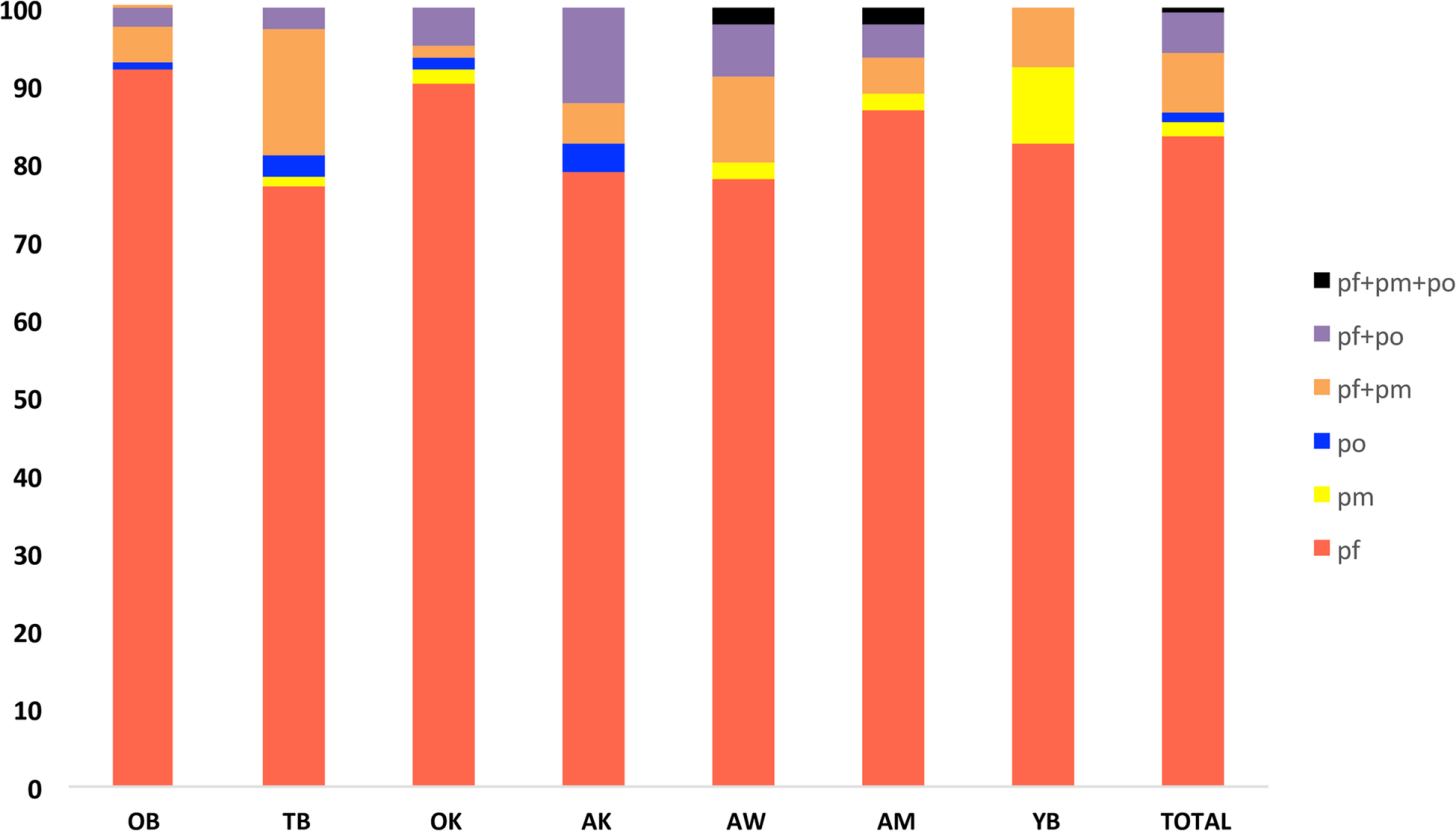
The distribution of PCR detectable *Plasmodium* species within the study sites. OB, Obosono; TB, Towoboase; OK, Okomfo; AK, Akwensu; AW, Awensu; AM, Amokope; YB, Yaw Boadi; mono species infection; pf: *Plasmodium falciparum* (red), pm: *Plasmodium malariae* (yellow), po: *Plasmodium ovale* (blue), multi species infection; pf + pm (orange); pf + po (purple); pf + pm + po (black). Prevalence is expressed as the % of total parasites identified in each site

**Fig. 5:**
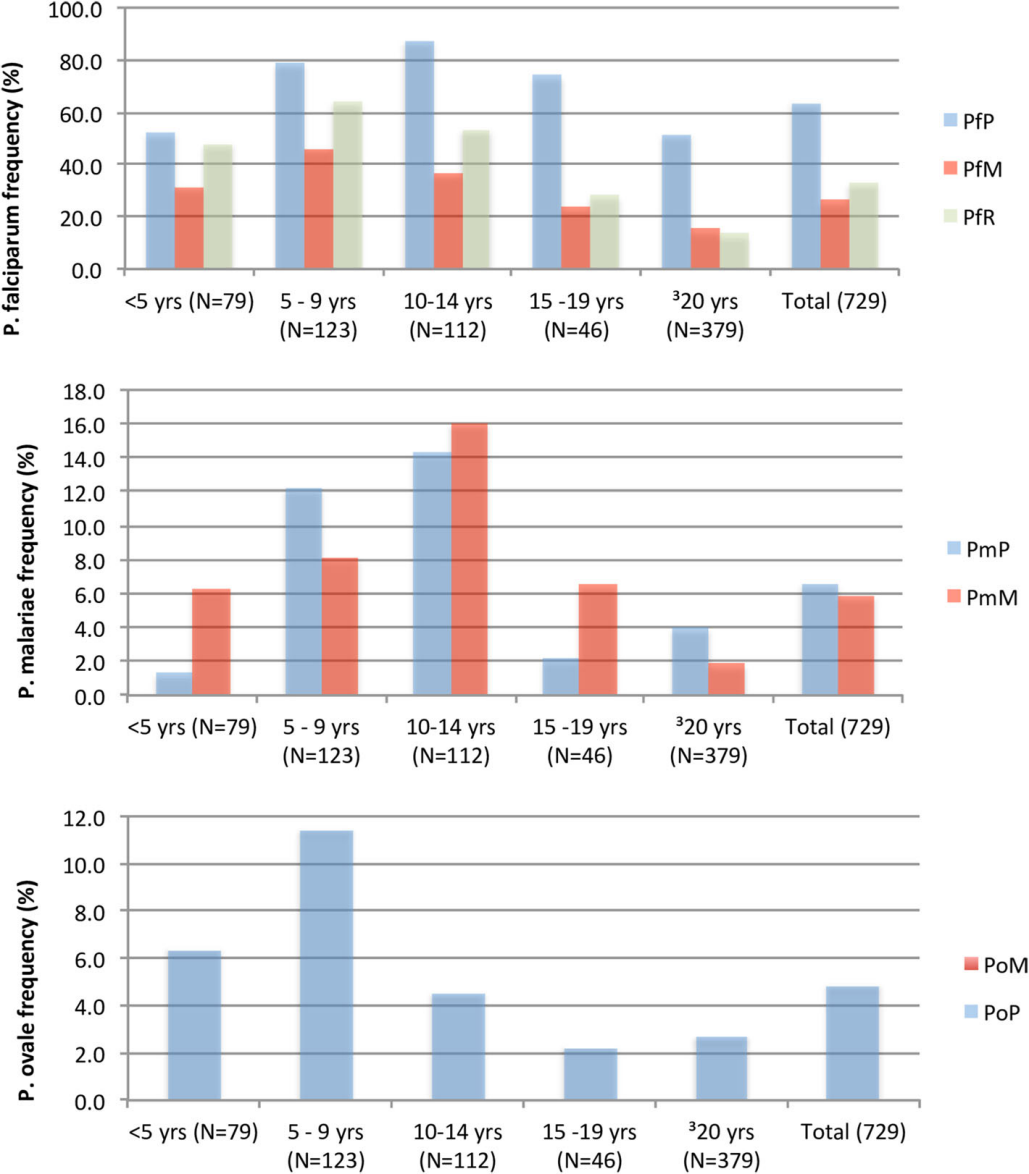
Comparison of diagnostic tools. A bar graph showing the prevalence of *P. falciparum* (Pf), *P. malariae* (Pm) and *P. ovale* (Po) as estimated by species specific PCR (P), microscopy (M) and RDT (R). values shown represent frequency expressed as a % of the total number of samples (729)

Parasite prevalence estimated by PCR across the five age groupings was significantly different for all the three *Plasmodium* species identified (Pearson Chi-Square 65.364, *p* = 0.000 (*P. falciparum*); 25.991, *p* = 0.000 (*P. malariae*); 16.311, *p* = 0.003 (*P. ovale*)). *Plasmodium falciparum* and *P. malariae* was least prevalent in the young children aged below 5 years old and highest in the older children aged between 9 and 14 years and *P. ovale* most prevalent in the children (5–9 years) and least in the two adult groups (2.2% in young adults and 2.7% in adults) (Table 3). However, across the seven sites, there was no significant difference in the distribution of *P. falciparum,* but significant differences were identified in the distribution of *P. malariae* (Pearson Chi-Square 16.435, *p* = 0.012) and *P. ovale* (Pearson Chi-Square 15.869, *p* = 0.014) (Fig. 4). The prevalence of parasite carriers was much higher when estimated by PCR compared to estimates by microscopy (Fig. 5). There were 62.4% (290/465) of the *P. falciparum* positive samples that were not detected as *P. falciparum* by microscopy. Molecular (PCR) identification increased the prevalence of *P. malariae* by 0.7% (5/729), however, *P. falciparum* was misdiagnosed as *P. malariae* in 7.7% (46/465) of the samples and *P. malariae* was misdiagnosed in 31.5% (15/48) of the samples and identified as *P. falciparum* by microscopy. The prevalence of *P. ovale* increased from 0 to 4.8% (35/729), similarly, 62.9% (22/35) of the *P. ovale* samples were misdiagnosed and identified as *P. falciparum* [86.3% (19/22)] or as *P. malariae* [13.6% (3/22)] by microscopy (Fig. 5).

## Discussions

In most malaria endemic countries in the World Health Organization Africa Region, *P. falciparum* is the major malaria parasite and represents close to 90% or more of the total malaria parasite population [1]. The 2018 world malaria report estimated *P. falciparum* to represent 100% of the total parasite population in Ghana [1]. Similarly, the National Malaria Control Program, Ghana uses parasitaemia in referring to the presence of malaria parasites, without the distinction between the various *Plasmodium* species [2, 28–30]. Co-infections of *P. malariae* or *P. ovale* with *P. falciparum* have been reported to lower the total parasite density of an infection [10] and also increase *P. falciparum* gametocyte prevalence [31]. Very few reports have determined the distribution or prevalence of non-*falciparum* parasites [5, 32], making this study very essential not only to fill a knowledge gap but also to inform policy on the need for sensitive tools to enable the identification of the under reported prevalence of *P. malariae* and *P. ovale* parasites.

The prevalence of *Plasmodium* carriers was generally high in this study with a PCR estimated average of 66% and a microscopy average of 32%. The increased prevalence determined by PCR is expected as microscopy is known to underestimate parasite prevalence, especially at low parasite densities [33]. The high prevalence of submicroscopic infections identified in this study as well as other studies [33–35] is a big challenge to malaria control in Ghana as the Test Treat and Track program is based on the microscopic detection of malaria parasites. This suggests that even if the program is 100% effective, malaria transmission will still be very high as half of the infected population is afebrile, thus not identified and treated. In this study, the prevalence *P. malariae* estimated by microscopy and PCR were similar, which was contrary to the differences between *P. falciparum* and *P. ovale* estimates, which were much higher by PCR when compared with microscopy. One main reason for the disparity was the high number of *P. falciparum* samples that were misclassified as *P. malariae* by the microscopists. Misclassification of *Plasmodium* species is particularly common in countries with a low prevalence of non-*falciparum* malaria primarily because of infrequent encounter by the microscopist and the subsequent low ability to identify morphological differences amongst the various *Plasmodium* species [36]. The World Health Organization recommends that uncomplicated infections harboring *P. falciparum* and *P. malariae* are treated with the same dose of artemisinin combination therapy [17] as such, a misclassification of *P. malariae* for *P. falciparum* and vice versa may not result in severe outcomes. However, due to the requirement of a radical cure agent such as Primaquine to clear *P. ovale* hypnozoites the treatment regimen is artemisinin combination therapy + Primaquine [17], thus misclassifications of *P. ovale* with *P. falciparum* could lead to severe *P. ovale* infections.

Children under the age of 5 years are considered to be the most vulnerable groups [1], however, in this study, children under 5 years did not have the highest prevalence of infection, although they had the highest parasite load. This observation supports the results from an earlier study conducted on adults and children with asymptomatic malaria infections living in Northern Ghana [35] as well as another study conducted on children from Ivory Coast and Mauritania [37]. A contrary finding has been reported in a study conducted on symptomatic malaria patients, where the parasite load in children under 5 was lower than those of older children [38]. The disparity in these findings could be due to the use of volunteers with different malaria status and suggests that young children with afebrile malaria infections may be able to withstand higher parasite densities compared with their symptomatic counterparts and older age groups. Older children aged between 10 and 14 years old were identified as having the highest prevalence of afebrile *Plasmodium* carriers, including both *P. falciparum* asexual and gametocyte as well as *P. malariae* parasite carriage. High gametocyte carriage in the group with the highest prevalence of both *P. falciparum* and *P. malariae* could be due to intra-host competition, which is known to enhance *P. falciparum* gametocyte production [31]. An earlier report on asymptomatic carriers in Kenya also identified children below the age of 15 years to harbor the highest levels of *P. malariae* than the older population, which is similar to the findings in this study [39]. All three groups of children in this study are more likely to enhance malaria transmission due to the high prevalence of microscopic densities of gametocytes carried by the group members. Although membrane feedings assays were not conducted in this study, other studies have found that high-density gametocyte infections contribute more to malaria transmission than low-density (sub microscopic) gametocyte infections [40].

In this study, age influenced the false positive RDT rates, with children having much higher false positive rates than the adult group. One main reason why the three groups of children exhibited high false positive rates could be because children generally have more incidents of clinical malaria as well as have higher density infections [41], which when treated would result in high levels of HRP2 antigen and a resultant longer duration of HRP2 antigen persistence that causes false positive RDT result [42]. Persistence of HRP2 antigen that is responsible for majority of false positive HRP2 RDT results is concentration dependent [43] and will take longer to clear after the treatment of high-density infections [44, 45]. False negative RDT results have also been associated with the presence of parasites with deletions in the Pfhrp2 gene [46, 47] or the presence of very high parasite densities that result in high concentrations of HRP2 antigen and a subsequent prozone effect [48]. False negative microscopy results are mainly due to the presence of submicroscopic densities of parasites, which are very common in asymptomatic infections [33]. Though there might be a possibility of false positive PCR results due to either contaminations during sample processing and amplification [49], nucleic acid amplification methods such as PCR has been recommended by the World Health Organization to be used to provide more accurate prevalence measurements during surveillance exercises as well as for malaria parasite detection in low transmission settings [50].

Even though afebrile parasite carriage was significantly affected by age, *Plasmodium* parasite carriage was similar in the seven closely linked communities. This suggests that although similar malaria control interventions would be effective in closely linked communities, interventions should be age group specific. There are a number of malaria control interventions that are targeted to protect children below the age of 5 years, one of the most vulnerable groups with respect to malaria from exposure to the malaria parasite [1]. The results from this study suggest that those interventions are effective as only a small fraction of the children below 5 years old living in the communities surveyed carried malaria parasites.

Previous reports on the distribution and prevalence of non-*falciparum* malaria parasites in Ghana vary from less than 1% to more than 12% across different regions of the country [5, 51, 52], with no reports of the presence of *P. vivax* in Ghana to date [5]. In order to obtain a more accurate measure of the nationwide distribution and prevalence of non-*falciparum* malaria in Ghana, it is necessary that nationwide molecular surveys of all human malaria parasites be conducted on a regular basis. This will help determine the geographic distribution and prevalence of non-*falciparum* malaria parasites, especially *P. ovale* and decide whether there is a need to implement a revision to the national malaria treatment policy to incorporate a treatment regimen for *P. ovale*, which is presently absent in Ghana.

## Conclusion

The high rate of misidentification of non-*falciparum* parasites and the total absence of detection of *P. ovale* by microscopy suggests that more sensitive malaria diagnostic tools including molecular assays are required to accurately determine the prevalence of carriers of non-*falciparum* parasites and low-density *P. falciparum* infections, especially during national surveillance exercises. Additionally, malaria control interventions targeting the non-*falciparum* species *P. malariae* and *P. ovale* parasites are needed.

## Supplementary information

**Additional file 1: Table S1.** List of primers and their properties

## Data Availability

The datasets used and/or analysed during the current study available from the corresponding author on reasonable request.

## Abbreviations

AM: Amokope
AN: Ankwensu
AW: Anwensu
DBS: Dried blood spot
OB: Obosono
OK: Okomfo
*P. falciparum*: *Plasmodium falciparum*
*P. malariae*: *Plasmodium malariae*
*P. ovale*: *Plasmodium ovale*
*P. vivax*: *Plasmodium vivax*
PBS: Phosphate buffered saline
PCR: Polymerase chain reaction
PD: Parasite density
RDT: Rapid diagnostic test
TO: Towoboase
YB: Yaw Boadi
