# Supplementary material for: Probing the composition of Plasmodium species contained in malaria infections in the Eastern region of Ghana
Source: BMC Public Health. 2019 Dec 2;19:1617. doi: 10.1186/s12889-019-7989-1 (PMC6889690; doi:10.1186/s12889-019-7989-1)
Supplement: Supplementary file 1 — Additional file 1: Table S1. List of primers and their properties [file 12889_2019_7989_MOESM1_ESM.docx]

Table S1. List of primers and their properties

| Species | Primer | Primer Sequence (5’-3’) | Size (bp) |
| --- | --- | --- | --- |
| *Plasmodium spp* | rPLU6 | TTA AAA TTG TTG CAG TTA AAA CG | 1050 |
|  | rPLU5 | CCT GTT GTT GCC TTA AAC TTC |  |
| *Plasmodium falciparum* | rFAl1 | TTA AAC TGG TTT GGG AAA ACC AAA TAT ATT | 205 |
|  | rFAL2 | ACA CAA TGA ACT CAA TCA TGA CTA CCC GTC |  |
| *Plasmodium malariae* | rMAL1 | ATA ACA TAG TTG TAC GTT AAG AAT AAC CGC | 144 |
|  | rMAL2 | AAA ATT CCC ATG CAT AAA AAA TTA TAC AAA |  |
| *Plasmodium ovale* | rOva1 | ATC TCT TTT GCT ATT TTT TAG TAT TGG AGA | 787 |
|  | rOva2 | GGA AAA GGA CAC ATT AAT TGT ATC CTA GTG |  |
